# The aetiologies of central nervous system infections in hospitalised Cambodian children

**DOI:** 10.1186/s12879-017-2915-6

**Published:** 2017-12-29

**Authors:** Paul Turner, Kuong Suy, Le Van Tan, Pora Sar, Thyl Miliya, Nguyen Thi Thu Hong, Vu Thi Ty Hang, Nguyen Thi Han Ny, Sona Soeng, Nicholas P. J. Day, H. Rogier van Doorn, Claudia Turner

**Affiliations:** 1 Cambodia Oxford Medical Research Unit, Angkor Hospital for Children, Siem Reap, Cambodia; 2 Mahidol-Oxford Tropical Medicine Research Unit, Faculty of Tropical Medicine, Mahidol University, Bangkok, Thailand; 3 Centre for Tropical Medicine and Global Health, Nuffield Department of Medicine, University of Oxford, Oxford, UK; 4 Oxford University Clinical Research Unit, Wellcome Trust Major Overseas Programme, in partnership with the Hospital for Tropical Diseases, Ho Chi Minh City, Vietnam

## Abstract

**Background:**

Central nervous system (CNS) infections are an important cause of childhood morbidity and mortality. The aetiologies of these potentially vaccine-preventable infections have not been well established in Cambodia.

**Methods:**

We did a one year prospective study of children hospitalised with suspected CNS infection at Angkor Hospital for Children, Siem Reap. Cerebrospinal fluid specimens (CSF) samples underwent culture, multiplex PCR and serological analysis to identify a range of bacterial and viral pathogens. Viral metagenomics was performed on a subset of pathogen negative specimens.

**Results:**

Between 1st October 2014 and 30th September 2015, 284 analysable patients were enrolled. The median patient age was 2.6 years; 62.0% were aged <5 years. CSF white blood cell count was ≥10 cells/μL in 116/272 (42.6%) cases. CNS infection was microbiologically confirmed in 55 children (19.3%). Enteroviruses (21/55), Japanese encephalitis virus (17/55), and *Streptococcus pneumoniae* (7/55) accounted for 45 (81.8%) of all pathogens identified. Of the pathogens detected, 74.5% (41/55) were viruses and 23.6% (13/55) were bacteria. The majority of patients were treated with ceftriaxone empirically. The case fatality rate was 2.5%.

**Conclusions:**

Enteroviruses, JEV and *S. pneumoniae* are the most frequently detected causes of CNS infection in hospitalised Cambodian children.

## Background

Meningitis and encephalitis are important causes of mortality and morbidity in children [1, 2]. These central nervous system (CNS) infections frequently result in neuro-developmental sequelae [3, 4]. The range of pathogens associated with CNS infections is broad with considerable geographic variability [5].

Data on aetiology are important, since several of the globally important pathogens are at least partially vaccine preventable: *Haemophilus influenzae* type b (Hib), *Neisseria meningitidis*, *Streptococcus pneumoniae,* measles, mumps, rubella, rabies, and Japanese encephalitis virus (JEV) [6–8]. However, there is a paucity of such data for many low and middle-income countries (LMICs) where the disease burden is highest [9–11]. In Cambodia, a low income Southeast Asian country, limited hospital-based surveillance suggests that Japanese encephalitis virus (JEV) is a major cause of acute encephalitis in children [12, 13]. A review of sentinel site surveillance data from 2006 to 8 found that JEV infection was detected in 19% of 586 meningoencephalitis cases, almost all in children aged ≤12 years [14, 15]. Unfortunately, data on non-JEV CNS infections in Cambodia remain scarce. However, a recent enterovirus 71 (EV71) outbreak infection highlighted the importance of this organism as a cause of severe CNS infection [16]. Hib and pneumococcal conjugate vaccine (PCV13) were introduced into the Cambodian nation immunisation schedule in 2010 and 2015, respectively. *H. influenzae* or *S. pneumoniae* were identified in a third (10/31) of hospitalised children with suspected CNS infection who were enrolled into a one year pre-Hib/PCV13 fever study [17].

## Methods

The aim of the current study was to provide an update on the aetiologies of CNS infections in Cambodian children aged 1 month to 15 years admitted to a paediatric referral hospital over a one year period.

### Study site

This one-year study was conducted at Angkor Hospital for Children (AHC), a non-governmental hospital located in Siem Reap, Northern Cambodia, and an associated Satellite Clinic (SC) at Sot Nikom District referral hospital, approximately 35 km from Siem Reap. AHC and SC provide secondary and tertiary level care to children aged 0–15 years, with no geographic patient restriction. There are approximately 180,000 patient episodes including 6000 hospital admissions per year, over the two sites.

### Study design and case definitions

Between 1st October 2014 and 30th September 2015, hospitalised children admitted to AHC or SC meeting the clinical case definition for suspected CNS infection were eligible for enrolment into the study (Table 1). Cases were identified by case note review of all new medical admissions during the preceding day, with receipt of CSF specimens in the microbiology laboratory acting as a secondary trigger for patient review.

**Table 1:** Study clinical case definition

Criterion^a^	Definition
Age	1 month – 15 years
Fever	≥38°C within 24 h of hospital admission
Clinical features	At least one of: • Stiff neck • Altered / reduced consciousness o V, P, or U on the AVPU score o <15 on the Glasgow Coma Score • Focal neurological symptoms / signs • Convulsions o Aged <6 months or ≥6 years: any seizure o Aged 6 months to <6 years: any focal or prolonged seizure OR ≥2 brief generalised seizures • Bulging fontanelle if <12 months of age • Irritability if <5 years of age • Headache • Prostration o Inability to drink or breast feed, or to remain sitting in a child otherwise able to sit • Petechial or purpuric rash
Laboratory investigation	Lumbar puncture performed, or actively planned at the time of assessment, by clinical team

^a^For study enrolment, all four criteria had to be met (age, fever, clinical, and laboratory)

### Clinical data and specimen collection

Clinical data regarding the features of the illness, household and environmental exposures, laboratory investigation and empiric treatment were obtained by parent/guardian interview plus review of the patient medical records and hospital electronic patient database. Enrolled cases were followed until discharge to capture treatment, complication, and outcome data. Laboratory investigations were requested at the discretion of the admitting clinician, with hospital guidelines recommending collection of blood and CSF cultures, along with blood for complete blood count (CBC), glucose, and electrolytes, in all suspected CNS infection cases. Study-specific training was provided to the AHC and SC clinicians at the beginning of the study to reinforce identification and appropriate investigation of suspected CNS infection.

### Laboratory assays

Except where noted, all study-related microbiology procedures were performed onsite at the AHC microbiology laboratory. The laboratory participates in the Pacific Paramedical Training Centre (PPTC) and World Health Organization (WHO) Invasive Bacterial Vaccine Preventable Diseases (IB-VPD) microbiology external quality assurance schemes.

Blood cultures were performed as previously described [18]. Briefly, 1-4 mL venous blood was inoculated into 20 mL culture medium (brain heart infusion broth (Oxoid, Basingstoke, UK) plus 0.05% sodium polyanethol sulphonate (Sigma-Aldrich, St. Louis, MO, USA) and the vented bottles were incubated at 37°C for up to seven days. Bottles were routinely sub-cultured onto solid media at 24 h and seven days, with additional sub-culture if turbidity was noted on daily inspection.

Cerebrospinal fluid specimens (3-4 mL) were routinely submitted for red and white blood cell counts (using a Fuchs-Rosenthal counting chamber with a Giemsa stained slide for differential white blood cell count), glucose and protein estimation (HumaStar 200 analyser; Human, Wiesbaden, Germany), and Gram stain. In addition, clinicians could request Ziehl–Neelsen or India ink staining if tuberculosis (TB) or cryptococcal infection was suspected, respectively. CSF was cultured onto 5% sheep blood agar, chocolate agar, MacConkey and Sabouraud agar plates (Oxoid, prepared in-house) for up to 48 h.

Bacterial isolates from blood and CSF cultures were identified using conventional microbiological techniques, including colonial morphology, growth requirements, Gram stain appearance, and confirmatory biochemical/serological tests. Specifically, *H. influenzae* was confirmed by X + V factor (Oxoid) dependent growth and serotyped by slide agglutination (BD Difco, Franklin Lakes, NJ, USA); *N. meningitidis* was confirmed by API NH profile (bioMerieux, Marcy L’ Etoile, France); and *S. pneumoniae* was confirmed by optochin disk susceptibility (Oxoid) and/or bile solubility.

Residual CSF specimens were stored in three aliquots at −80°C for molecular and serological analyses. Two CSF aliquots were used for bacterial (*H. influenzae* type b, *N. meningitidis, S. pneumoniae*) and viral (enteroviruses (EV), herpes simplex virus 1 and 2 (HSV), mumps virus (MV), parechoviruses (PV), varicella zoster virus (VZV)) pathogen detection by multiplex PCR assays (Fast-Track Diagnostics (FTD), Sliema, Malta). Briefly, nucleic acids were extracted from 200 μL thawed CSF using the QIAamp MinElute Virus Spin Kit (Qiagen, Hilden, Germany), with a final elution volume of 60 μL. Ten microliters of extracted nucleic acid solution was used as the template in each reaction of the FTD Bacterial Meningitis and Viral Meningitis multiplex PCRs which were run on a CFX96 real-time PCR instrument (Bio-Rad, Hercules, CA, USA), following the manufacturer’s instructions. Specimens were determined to be pathogen positive or negative based on kit criteria. PCR runs were repeated in the event of quality control failure (i.e. failure of positive control amplification or amplification in negative control wells). Failure of extraction control amplification resulted in re-extraction of the CSF specimen and repeat PCR testing, if sufficient CSF was available. The final CSF aliquot was sent to the Oxford University Clinical Research Unit (OUCRU), Ho Chi Minh City, Vietnam for identification of JEV infection by a capture immunoglobulin M enzyme-linked immunosorbent assay (IgM ELISA) assay to determine dengue virus and JEV-specific IgM (Venture Technologies, Sarawak, Malaysia), as previously described [19, 20].

Once the core study investigations had been completed, pathogen-negative purulent CSF specimens from patients who had been unwell for five days or less were submitted to a viral metagenomics pipeline, using a non-ribosomal random PCR and Illumina Miseq-based assay as described previously [21, 22]. Any evidence of viral infection, as indicated by metagenomic analysis, was subsequently confirmed by specific PCR [23].

### Data analysis

Clinical cases were characterised as confirmed, probable, or suspected CNS infection based on laboratory criteria (Table 2 [20, 24–26]). Those patients in whom a lumbar puncture was attempted but was unsuccessful, or was deemed unnecessary and cancelled post-enrolment, were classified as suspected. Children were grouped by age: 1–11 months (infants); 1–4 years (young children); and 5–15 years (older children).

**Table 2:** Final case categorisation, based on laboratory features

CNS infection category	Features (in addition to meeting clinical case definition)
Suspected	Non-purulent CSF *AND* absence of identifiable pathogens by culture, PCR, or serology
Probable	Purulent CSF (WBC ≥100 cells/μL *OR* [WBC 10–99 cells/μL *AND* glucose <2.2 mmol/L or protein >1.0 g/L]) *AND* absence of identifiable pathogens by culture, Gram stain, PCR, or serology^a^ *OR* Abnormal CSF (WBC 10–99 cells/μL *AND* normal protein/glucose) *AND* positive blood culture and/or positive CSF Gram stain
Confirmed	Pathogen detected in CSF by culture and/or PCR *OR* Positive serology in CSF *OR* Positive blood culture *AND* purulent CSF *OR* Purulent CSF *AND* positive CSF Gram stain

^a^Pathogen-negative probable cases were re-categorised as suspected cases if laboratory testing was incomplete

Data were analysed using the R statistical package version 3.3.0 [27]. Non-normally distributed numeric variables were described by the median and range. Comparisons between age groups were made using the Kruskal-Wallis test (numeric variables) or the Chi-squared test for trend (categorical variables). Statistical significance was indicated by two-sided *p*-values of <0.05.

## Results

### Enrolment

**Fig. 1:**
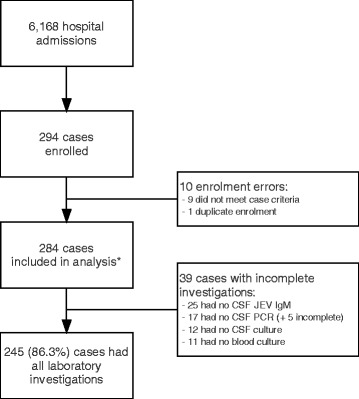
Study enrolment flowchart. *Including one culture-confirmed meningitis who did not fully meet the clinical case definition

In total, there were 6168 patient admissions to AHC (*n* = 4439) and SC (*n* = 1729) between 1st October 2014 and 30th September 2015. From these admissions, 294 (4.8%) children were enrolled into the study and 284 were included in the analyses below (Fig. 1).

### Clinical findings

**Fig. 2:**
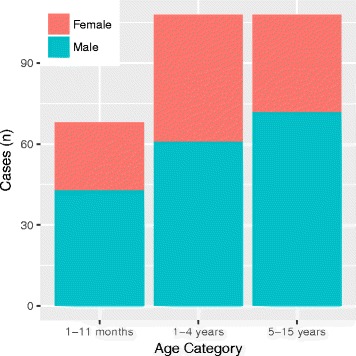
Age distribution of 284 hospitalised children meeting the clinical case definition for CNS infection

The median age at admission was 2.6 years and 62% (176/284) were aged <5 years. There were more male than female children (176/284; 62.0%) but there were no differences in the gender distribution by age group (Fig. 2; *p* = 0.3). Almost all children were admitted to AHC/SC directly from home (248/284; 87.3%), following a median of 4 days of symptoms (IQR 3–6). Older children had a longer median symptom duration compared to the younger age groups (5 days versus 3 (1-4y) and 4 (1-11 m); *p* < 0.0001). Three children (3/283; 1.1%) reported symptoms for at least 4 weeks prior to admission. Almost half of the children (130/280; 46.4%) had been given medication in the week prior to hospital admission: this was more common in older children compared with younger age groups (64.8% versus 37.5% (1-4y) and 30.9% (1-11 m); *p* = 0.003). In most cases (106/130; 81.5%), the details of the medication were unknown. However, antibiotic use was definitively reported in 18.5% (24/130). Pre-existing HIV infection was known in three (1.1%) children. Treatment for TB was documented in 20 children: 16 reported previous treatment and four were receiving treatment at the time of admission. Immunisation record cards were available for review in 60 (21.2%) cases. All 60 had received BCG at birth and 53 (88.3%) had received at least one dose of Hib vaccine. Only three (5%) children had received either JEV or PCV13 vaccines. Around half of the children had recent contact with either a domestic (144/282; 51.1%) or livestock (135/281; 48.0%) animal. Major clinical features are summarised in Table 3.

**Table 3:** Presenting features of 284 hospitalised children meeting the clinical case definition for CNS infection

Clinical feature	1-11 m	1-4y	5-15y	Overall	*P*-value
	(*n* = 68)	(*n* = 108)	(*n* = 108)	(*n* = 284)	
History					
Symptom duration (days), median (range)	4 (1–30)	3 (1–50)	5 (1–30)	4 (1–50)	<0.0001
Fever, n (%)	68 (100)	108 (100)	108 (100)	284 (100)	–
Respiratory symptoms^a^, n (%)	49 (72.1)	61 (56.5)	37/107 (34.6)	147/283 (51.9)	<0.0001
Vomiting, n (%)	33 (48.5)	37 (34.3)	81 (75.0)	151 (53.2)	<0.0001
Diarrhoea, n (%)	25 (36.8)	30 (27.8)	18 (16.7)	73 (25.7)	0.002
Reduced feeding / eating & drinking, n (%)	40 (58.8)	62/105 (59.0)	73 (67.7)	175/281 (62.3)	0.0007
Lethargic^b^, n (%)	34/66 (51.5)	45/102 (44.1)	63/107 (58.9)	142/275 (51.6)	0.2
Headache, n (%)	NA	NA	100/107 (93.5)	–	–
Photophobia, n (%)	NA	NA	9/104 (8.7)	–	–
Seizures, n (%)	47 (69.1)	86 (79.6)	18 (16.7)	151 (53.2)	<0.0001
*Generalised seizure*	43 (63.2)	79 (73.1)	16 (14.8)	138 (48.6)	–
*Focal seizure*	4 (5.9)	6 (5.6)	2 (1.9)	12 (4.2)	–
*Unknown seizure type*	0 (0)	1 (0.9)	0 (0)	1 (0.4)	–
Medication in week prior to admission, n (%)	21 (30.9)	39/104 (37.5)	70 (64.8)	130/280 (46.4)	0.003
* Antibiotic* ^c^	6 (8.8)	7/104 (6.7)	11 (10.2)	24/280 (8.6)	–
*Unknown medication*	15 (22.1)	32/104 (30.8)	59 (54.6)	106/280 (37.9)	–
Physical findings					
Temperature (°C), median (range)	38.7 (36.5–40.3)	39.0 (38.0–40.8)	38.7 (38.0–42.0)	38.9 (36.5–39.4)	0.3
Reduced conscious level^d^, n (%)	3/50 (6.0)	12/89 (13.5)	11/104 (10.6)	26/243 (10.7)	0.5
Bulging anterior fontanelle, n (%)	17/64 (26.6)	NA	NA	NA	–
Neck stiffness / positive Kernig’s sign, n (%)	6/64 (9.4)	6/93 (6.5)	31/98 (31.6)	43/255 (16.9)	0.01
Limb weakness, n (%)	2 (2.9)	1 (0.9)	11 (10.2)	14/284 (4.9)	0.01
Rash, n (%)	5/66 (7.6)	2/102 (0.02)	3/102 (2.9)	10/270	0.2

*NA* not age applicable

^a^Any of: cough, dyspnoea, rhinorrhoea, earache

^b^Difficult to wake up or more sleepy than normal

^c^Amoxicillin (14); ceftriaxone (5); ampicillin (2); ciprofloxacin (2); ceftriaxone + gentamicin (1)

^d^Admission GCS < 15/15 or V, P, or U on AVPU scale

Lumbar puncture was successfully performed in 272 cases. Of these, 116 (42.6%) had a CSF white blood cell (WBC) count of ≥10 cells/μL. Cases were more likely to be classified as suspected (i.e. non-purulent CSF and no pathogen detected) in infants (82.3%) and young children (79.6%) compared with older children (47.2%) (*p* < 0.0001; Table 4).

**Table 4:** Treatment and outcomes of 284 hospitalised children meeting the clinical case definition for CNS infection

Feature	1-11 m	1-4y	5-15y	Overall	*P*-value
	(*n* = 68)	(*n* = 108)	(*n* = 108)	(*n* = 284)	
Admission duration (days), median (range)	5 (1–49)	4 (1–23)	7 (0–68)	5 (0–68)	<0.0001
Empiric antibiotic treatment^a^, n (%)					
*Ampicillin*	1 (1.5)	2 (1.9)	0 (0)	3 (1.1)	0.3
*Ceftriaxone*	41 (60.3)	67 (62.0)	92 (85.2)	200 (70.4)	0.0001
*Imipenem / meropenem*	0 (0)	1 (0.9)	0 (0)	1 (0.4)	0.9
*Other single antibiotic drug*	1 (1.5)	5 (4.6)	2 (1.9)	8 (2.8)	0.9
*Multiple empiric antibiotics*	6 (8.8)	9 (8.3)	9 (8.3)	24 (8.5)	0.9
*No empiric antibiotics*	19 (27.9)	24 (22.2)	5 (4.6)	48 (16.9)	<0.0001
No antibiotic given, n (%)	16 (23.5)	22 (20.4)	2 (1.9)	40 (14.1)	<0.0001
Dexamethasone, n (%)	14 (20.6)	13 (12.0)	44/107 (41.1)	71/283 (25.1)	0.0003
ICU care, n (%)	25 (36.8)	22 (20.4)	20 (18.5)	67 (23.6)	0.009
Outcome, n (%)					
*Home*	64 (94.1)	102 (94.4)	103 (95.4)	269 (94.7)	0.7
*Death (incl. Home to die)* ^b^	1 (1.5)	2 (1.9)	4 (3.7)	7 (2.5)	0.3
*Unknown (transfer / LAMA* ^c^ *)*	3 (4.4)	4 (3.7)	1 (0.9)	8 (2.8)	0.3
CNS infection case classification, n (%)					
*Confirmed*	11 (16.2)	20 (18.5)	24 (22.2)	55 (19.3)	0.3
*Probable*	1 (1.5)	2 (1.9)	33 (30.6)	36 (12.7)	<0.0001
*Suspected*	56 (82.3)	86 (79.6)	51 (47.2)	193 (68.0)	<0.0001

^a^Defined as treatment initiated within the first 48 h of hospital admission

^b^One patient was taken home when condition was deemed hopeless

^c^Left against medical advice

Ceftriaxone was the most commonly used empiric antibiotic treatment, prescribed for 200/236 (84.7%) children who received empiric treatment. No antibiotic was prescribed in 40 cases, mostly infants and young children (38/40; 95.0%). Children with a CSF WBC of <10 cells/ μL were less likely to be prescribed an antibiotic compared to those with higher CSF WBCs (31/156 (19.9%) versus 6/116 (5.2%); *p* = 0.0005). Dexamethasone was given as adjunctive treatment in 71 children (25.1%), predominantly in older children (41.1% versus 12.0% (1-4y) and 20.6% (1-11 m); *p* = 0.0003). A quarter (67/288; 23.1%) of children required management in the intensive care unit: this was more frequent in infants compared to the older age groups (36.8% versus 20.4% (1-4y) and 18.5% (5-15y); *p* = 0.09). The overall acute mortality was 2.5% (7/284). Treatment and outcomes are summarised in Table 4.

### Pathogen detection

Overall, a pathogen was identified in the blood or CSF of 20.8% (59/284) children. A single pathogen was identified per child. A pathogen was detected in 47/116 (40.5%) children with CSF WBC ≥10/μL compared to 11/156 (7.1%) cases with CSF WBC <10 cells/μL (*p* < 0.0001).

Fifty-five children were included in the confirmed CNS infection category (Tables 4 & 5). Enteroviruses (21/55), JEV (17/55), and *S. pneumoniae* (7/55) accounted for 81.8% of all pathogens identified. Of the pathogens detected, 74.5% (41/55) were viruses and 23.6% (13/55) were bacteria. There was a single case of cryptococcal meningitis in an HIV-positive child.

**Table 5:** Pathogens identified in 55 children with laboratory-confirmed CNS infection

Pathogen, n (%)	1-11 m (*n* = 11)	1-4y (*n* = 20)	5-15y (*n* = 24)	Overall (*n* = 55)	*P*-value
Enterovirus^a^	2 (18.2)	8 (40.0)	11 (45.8)	21 (38.3)	0.1
Japanese encephalitis virus	1 (9.1)	7 (35.0)	9 (37.5)	17 (31.0)	0.1
*Streptococcus pneumoniae*	3 (27.2)	3 (15.0)	1 (4.2)	7 (12.7)	0.05
*Escherichia coli*	2 (18.2)	0 (0)	0 (0)	2 (3.6)	–
Mumps virus	0 (0)	0 (0)	2 (8.3)	2 (3.6)	–
*Neisseria meningitidis*	2 (18.2)	0 (0)	0 (0)	2 (3.6)	–
*Cryptococcus neoformans*	0 (0)	0 (0)	1 (4.2)	1 (1.8)	–
*Haemophilus influenzae* type b	1 (9.1)	0 (0)	0 (0)	1 (1.8)	–
Herpes simplex virus	0 (0)	1 (5.0)	0 (0)	1 (1.8)	–
*Staphylococcus aureus*	0 (0)	1 (5.0)	0 (0)	1 (1.8)	–
Parechovirus	0 (0)	0 (0)	0 (0)	0 (0)	–
Varicella zoster virus	0 (0)	0 (0)	0 (0)	0 (0)	–

^a^Another 4 children (all 5-15y) were enterovirus positive on deep sequencing (not part of the core diagnostic strategy)

Blood cultures were positive in four suspected CNS infection cases, one each of *Burkholderia pseudomallei, S. pneumoniae* (in a child from whom a CSF specimen was not obtained), *Streptococcus pyogenes,* and *Streptococcus salivarius* (this isolate was deemed to be of uncertain clinical significance, in the context of the patient presentation)*.*

PCR or serology identified the majority of pathogens: 55.9% (33/59) were identified by PCR and 28.8% (17/59) by serology. Only 15.3% (9/59) were identified by culture only (including two pneumococcal blood culture positive cases where CSF was not available for PCR testing). Four bacterial meningitis cases were PCR positive but culture negative (1 *N. meningitidis* and 3 *S. pneumoniae*) whilst the others were all culture and PCR positive for the same species. Two of these children had received an unknown medication prior to hospital presentation. Of the 24 children who had definitely received an antibiotic prior to lumbar puncture, three were EV PCR positive, one was JEV IgM positive, and another had *Streptococcus pneumoniae* isolated from blood culture. In the latter case, CSF was microscopically abnormal but culture negative with insufficient material for PCR testing.

A pathogen was identified in three of the seven children who died: *Cryptococcus neoformans* (1; confirmed case in an HIV positive child)*, B. pseudomallei* (1; suspected case), JEV (1; confirmed case).

**Fig. 3:**
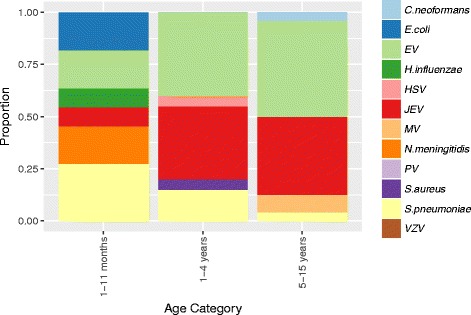
Pathogen distribution by age group in 55 children with laboratory-confirmed CNS infection. EV: enterovirus; HSV: herpes simplex virus; JEV: Japanese encephalitis virus; MV: mumps virus; PV: parechovirus; VZV: varicella zoster virus

Whilst there were insufficient numbers to determine clear associations between pathogens and age, both EV and JEV were less frequently detected in infants compared to older children with the converse was true for *S. pneumoniae* (Fig. 3). Pathogens were detected in the CSF in four young children diagnosed as having febrile convulsions: JEV (2), EV (1), and HSV (1).

Tuberculous meningitis was clinically suspected in eight cases; Ziehl-Neelsen stain results were negative in the two specimens it was requested on. JEV IgM was detected in the CSF of three of the clinically suspected TB cases and the others were pathogen negative. Following CT scan, two patients were diagnosed with brain tumours and another with a cerebral abscess. One child was given a clinical diagnosis of rabies and died in hospital.

### Deep sequencing results

Enterovirus reads were detected by deep sequencing in 4/19 (21.1%) probable CNS infection cases. In two cases, the viruses were further characterised as genotypes within the *Enterovirus B* species (Echovirus E6 and 30). Whilst the original viral multiplex PCR was negative in these cases, subsequent enterovirus PCR testing was positive in all. Additionally, HIV reads were detected in one child with known HIV infection.

## Discussion

This study has demonstrated the predominant pathogens associated with CNS infections in hospitalised Cambodian children. Enteroviruses, JEV, and *S. pneumoniae* were responsible for a significant proportion of pathogen-positive cases. The detection of enterovirus in four patients, who were originally PCR negative, by viral metagenomics analysis reveals the potential performance limitations of commercial multiplex PCR assays. However, it also alludes to the potential diagnostic utility of next generation sequencing as a single pan-pathogen assay. Despite the use of culture, serology, PCR and metagenomic sequencing, a pathogen was not established in >50% of cases with a raised CSF white cell count, highlighting the difficulty in confirming aetiology in paediatric meningo-encephalitis cases [2].

As expected, due to routine immunisation, the numbers of *H. influenzae* type b meningitis cases were small. It is expected that PCV13 vaccine introduction will have a similar effect on pneumococcal meningitis over the next few years. Pre-PCV13 data from AHC has confirmed that 88–92% of invasive pneumococcal disease in Cambodian children is caused by serotypes covered by the vaccine [28, 29]. The inclusion of JEV vaccine into the national immunisation schedule is imminent. On-going monitoring of CNS infections via national surveillance will be critical to monitor the impact of these vaccines and to detect the emergence of new pathogens.

Importantly, most of the pathogens would have been missed by a culture-only diagnostic approach: less than 20% of pathogens were identified by culture only. All the confirmed bacterial infections and 85% (35/41) of the confirmed viral infections had been started on empiric antibiotic treatment. Rapid availability of the PCR / serology results could have resulted in earlier cessation of unnecessary antibiotic treatment in children with viral infections. This finding also has financial implications for laboratories in LMICs, as robust commercially available nucleic acid extraction and multiplex PCR kits remain expensive. The current absence of a well-validated commercial IgM ELISA capable of distinguishing between acute dengue and JEV infection is also troublesome [30, 31].

The results are similar to comparable studies in the region. Enteroviruses and *S. pneumoniae* were found to be the most frequent causes of meningitis in Fijian children over a three-year period [32]. *Streptococcus pneumoniae* and *H. influenzae* type b were co-dominant causes of meningitis in Vietnamese children [33], whereas JE and enteroviruses were frequently identified in paediatric encephalitis cases [20]. In contrast, Laman and colleagues frequently identified human herpes viruses (HHV6/7) in CSF specimens from hospitalised children in Papua New Guinea, perhaps due to reactivation as a consequence of malaria infection [34]. *Streptococcus suis* was not identified by culture in the current study, although this was not expected to be a major pathogen given the young age of the patients [33, 35].

There were several limitations to the study. The study was conducted at a single site over one year. The fragmented nature of the Cambodian health system made it impossible to estimate disease incidence. Extrapolation of findings to a wider geographic area must be done cautiously. However, the fact that the hospital provides free treatment, including laboratory investigation, may have reduced patient selection bias. Enterovirus meningitis occurs as part of larger enteroviral outbreaks [36]. A multi-year study, with more detailed characterisation of the EV group, would have permitted more accurate determination of the relative importance of these viruses. The panel of organisms included was somewhat limited. For viruses, inclusion of measles, rabies, and rubella PCR would have been desirable and testing of supporting specimens (e.g. serum / urine (flaviruses), rectal / throat swabs (enteroviruses)) may have increased the diagnostic yield. Identification of tuberculous meningitis (TBM) was sub-optimal: neither molecular (e.g. GeneXpert) or culture was possible during the study period. It is possible that cases of TBM were missed, although Vietnamese children with probable or definite TBM had longer duration of symptoms before hospital review (median 19.5 days) than were seen in the current study [37]. Testing for *Rickettsia* spp. or *Orientia tsutsugamushi* was not performed. A recent study has demonstrated the relative importance of these organisms as causes of CNS infections in Laos [38]. However, the majority of Lao cases were in adult patients. The previous AHC fever study identified *O. tsutsugamushi* and *R. typhi* in 7.8% and 2.2% of febrile admissions, respectively. These limitations suggest that, in addition to virus detection, it would have been desirable to have included a bacterial metagenomics approach in pathogen negative cases. Finally, the low case fatality rate plus the high proportion of normal WBC CSF specimens suggests that the clinical case definition was poorly specific. Children with simple febrile seizures were likely over-represented as a result of the difficulties in accurately characterising pre-hospital seizures. However, despite these limitations the study has clearly demonstrated the major pathogens in this patient group and has highlighted the range of diagnostic modalities required to accurately characterise the aetiology of CNS infections in SE Asia.

## Conclusions

Enteroviruses, JEV and *S. pneumoniae* are the most frequently detected causes of CNS infection in hospitalised Cambodian children. This finding should be reflected in laboratory testing strategies and patient management protocols.

## Abbreviations

AHC: Angkor Hospital for Children
AVPU: Alert, Voice, Pain, Unresponsive scale
CBC: Complete Blood Count
CNS: Central Nervous System
CSF: Cerebrospinal Fluid
ELISA: Enzyme-Linked Immunosorbent Assay
EV(71): Enterovirus (71)
FTD: Fast-Track Diagnostics
GCS: Glasgow Coma Scale
HHV: Human Herpes Virus
Hib: *Haemophilus influenzae* type b
HIV: Human Immunodeficiency Virus
HSV: Herpes Simplex Virus
IB-VPD: Invasive Bacterial Vaccine Preventable Diseases
ICU: Intensive Care Unit
IgM: Immunoglobulin M
JEV: Japanese Encephalitis Virus
LAMA: Left Against Medical Advice
LMIC: Low and Middle-Income Country
m: Months of age
MV: Mumps Virus
OUCRU: Oxford University Clinical Research Unit
PCR: Polymerase Chain Reaction
PCV (13): Pneumococcal Conjugate Vaccine (13-valent)
PPTC: Pacific Paramedical Training Centre
PV: Parechovirus
SC: Satellite Clinic
SE: South East
TB(M): Tuberculosis (meningitis)
VZV: Varicella Zoster Virus
WBC: White Blood Cell
WHO: World Health Organization
y: Years of age
